# Quantum Control by Few-Cycles Pulses: The Two-Level Problem

**DOI:** 10.3390/e25020212

**Published:** 2023-01-22

**Authors:** François Peyraut, Frédéric Holweck, Stéphane Guérin

**Affiliations:** 1 ICB, UMR 6303, CNRS, University Bourgogne Franche-Comté, UTBM, 90010 Belfort, France; 2 Department of Mathematics and Statistics, Auburn University, Auburn, AL 36849, USA; 3 ICB, UMR 6303, CNRS, University of Bourgogne, 21000 Dijon, France

**Keywords:** quantum control, quantum system driven by an external field, adiabatic passage, adiabatic Floquet theory

## Abstract

We investigate the problem of population transfer in a two-states system driven by an external electromagnetic field featuring a few cycles, until the extreme limit of two or one cycle. Taking the physical constraint of zero-area total field into account, we determine strategies leading to ultrahigh-fidelity population transfer despite the failure of the rotating wave approximation. We specifically implement adiabatic passage based on adiabatic Floquet theory for a number of cycles as low as 2.5 cycles, finding and making the dynamics follow an adiabatic trajectory connecting the initial and targeted states. Nonadiabatic strategies with shaped or chirped pulses, extending the π-pulse regime to two- or single-cycle pulses, are also derived.

## 1. Introduction

Quantum (or coherent) control aims at developing methods for manipulating quantum dynamical processes at the atomic or molecular scale by shaped external electromagnetic fields [1,2]. It can be conceptually formulated as the design of the field constituents (phase, amplitude, and polarization) driving the quantum system, such as atom, molecule [3], photonic field [4], nanostructures (quantum dot) and plasmonic field [5], Bose–Einstein condensate [6,7], superconducting circuit [8,9], …, from an initial state to a target state featuring the desired outcome. The latter can be a single bare state of the system, a coherent superposition of bare states, or the full propagator (i.e., for any initial state) as it is the case in quantum computation for which quantum gates are devised [10,11,12,13]. For instance, applications to quantum information target the superposition of a few states (qubit and its generalization) while the control of chemistry deals with wave packets made up of many states, such as, e.g., an aligned or oriented molecule corresponding to a superposition of many rotational states [14,15]. Various techniques have been proposed and implemented: Adiabatic passage [16,17] and adiabatic Floquet theory [18], composite pulses [19,20,21], optimal control [22,23,24], robust single-shot [25] and optimal [26] shaped pulses as a variant of shortcut to adiabaticity [27,28], and reinforcement learning [29,30].

Ultrashort laser pulses that last only a few optical cycles have been transformative tools for studying and manipulating light–matter interactions [31]. They allow in particular high nonlinear effects for strong-field processes in the subfemtosecond extreme ultraviolet/X-ray regime [32]. A different single-cycle THz regime has been used to experimentally demonstrate enhanced orientation of molecules [33]. Such single-cycle THz pulses have been investigated for the control of the rotational wavepackets, resulting in long-lasting orientation [34] and orientational quantum revivals [35].

The goal in this paper is twofold: We implement adiabatic Floquet theory [18] and explore its limit for few cycles. We show that it remarkably applies for a number of cycles as low as 2.5 cycles. In particular, one succeeds to realize an *ultrahigh-fidelity* population transfer (defined by a deviation from the perfect transfer below or of the order $10^{-4}$) by an appropriate design based on parallel adiabatic passage. Its obstruction for a lower number of cycles is due to the presence of nonadiabatic terms emerging from the counter-rotating term beyond the rotating wave approximation (RWA) [36]. We also investigate how one can control complete population transfer when a two-level system is driven by a shaped and chirped few-cycles pulse, typically N<3, taking the constraint of a zero field area into account. One generalizes the two standard techniques in this context, adiabatic passage and π-pulse method [36]. The latter is of importance as it is known to achieve the so-called quantum speed limit in RWA [24]. The former is known to induce robust transitions [17]. We achieve complete transfer for a single cycle N=1 pulsed laser featuring an appropriate chirped (time-dependent) frequency around the resonance.

After having defined the model in Section 2, we apply adiabatic Floquet theory in Section 3. In Section 4, we introduce the notion of generalized π-pulse in the context of few cycles. A generalized π-pulse consists in expanding the Rabi frequency as a Fourier series and selecting the appropriate modes leading to resonance and allowing complete transfer. This gives rise to specific strategies depending on the modes chosen in the Fourier expansion and on the chirping of the frequency. Results from numerical optimization are presented and connected to the preceding strategies in Section 6. We finally conclude in Section 7.

## 2. The Model with a Few-Cycle Pulse

We consider the two-level system {|−〉,|+〉} driven by a few-cycle pulse (in units such that ℏ=1)

(1) $$\begin{array}{cc} H= & \left[\begin{array}{cc} -\omega_{0}/2 & \Omega (t)\cos\phi (t)\\ \Omega (t)\cos\phi (t) & \omega_{0}/2 \end{array}\right], \end{array}$$

where Ω(t) is the Rabi frequency defined as $\Omega \left(t\right)=-\mu_{01}E\left(t\right)/\hbar$ (considered positive without loss of generality) with the dipole moment $\mu_{01}$ coupling the two states; $\omega_{0}>0$, and ϕ(t) are the Bohr frequency and the instantaneous phase, respectively. The effective (instantaneous) frequency $\omega \left(t\right):=\dot{\phi }\left(t\right)$ can be modified at will through the phase ϕ(t).

One can exhibit an instantaneous relative phase $\phi_{0}\left(t\right)$ with respect to the Bohr frequency:

(2) $$\phi \left(t\right)=\omega_{0}\left(t-t_{i}\right)-\phi_{0}\left(t\right).$$

and the corresponding detuning Δ:

(3) $$\Delta \left(t\right):=\omega_{0}-\omega \left(t\right)={\dot{\phi }}_{0}\left(t\right),\;\phi_{0}\left(t\right)=\int_{t_{i}}^{t}\Delta \left(s\right)ds+\phi_{0,i}$$

with $t_{i}=-T/2$ the initial time, $t_{f}=-t_{i}$ the final time with full duration $T=t_{f}-t_{i}$, and the initial phase $\phi_{0,i}$. We will restrict our study to the near-resonant case $\dot{\phi }\ll \omega_{0}$ for the ease of implementation. The number N of oscillations in the pulse (with respect to the resonant frequency $\omega_{0}$ in this near-resonant case) is such that

(4) $$N=T\frac{\omega_{0}}{2\pi }.$$

The physical implementation of the few-cycle field imposes its zero time-integrated area [37]:

(5) $$\int_{t_{i}}^{t_{f}}\Omega \left(t\right)\cos\left(\phi \left(t\right)\right)dt=0,$$

which will be imposed for all the derived fields in this work. After application of the resonant (or rotating wave) transformation:

(6) $$\begin{array}{cc} R= & \left[\begin{array}{cc} 1 & 0\\ 0 & e^{-i\phi (t)} \end{array}\right], \end{array}$$

we obtain (where we have for convenience shifted the origin of energy such that a traceless matrix is derived)

(7) $$\begin{array}{c} \tilde{H}=R^{†}HR-iR^{†}\frac{\partial R}{\partial t}=\frac{1}{2}\left[\begin{array}{cc} -\Delta (t) & \Omega (t)\\ \Omega (t) & \Delta (t) \end{array}\right]+\frac{\Omega (t)}{2}\left[\begin{array}{cc} 0 & e^{-2i\phi (t)}\\ e^{2i\phi (t)} & 0 \end{array}\right]. \end{array}$$

We notice that the rotating wave transformation changes not the amplitude of the solution but its phase. The first matrix corresponds to the standard (resonant) RWA. The second matrix defines the counter-rotating term. The full Hamiltonian (7) does not feature any approximation in the two-state model. It is well known that the counter-rotating Hamiltonian can be neglected in the limit $\Omega \ll \omega_{0}$, since, in this case, this term features fast oscillations that have a weak effect compared to the resonant, non-oscillatory, RWA Hamiltonian.

In the frame of the Floquet theory (see Appendix A and the next section), the RWA Hamiltonian characterizes the interaction between the states |−,0〉 and |+,−1〉, corresponding to the bare states of the system dressed with 0 photon and −1 photon, respectively, which are near degenerate (exactly degenerate on exact resonance). This means that the system can emit a photon when its state is transferred from state |+〉 to state |−〉 (or reciprocally can absorb a photon from state |−〉 to state |+〉).

Taking the simple example of a resonant (Δ=0) and flat (constant) pulse of amplitude $\Omega_{0}$ and duration *T*, the well-known π-pulse condition $T\Omega_{0}=\pi$ in (4) gives $\Omega_{0}/\omega_{0}=1/\left(2N\right)$. Thus, the constraint of a few oscillations in the field, typically N<3, implies that $\Omega_{0}$, while still smaller than $\omega_{0}$, becomes of the same order. This condition clearly *prevents to apply the resonant approximation*. This can be reformulated as follows: a small number of cycles in the pulse corresponds to a broadening of the spectrum of the field instantaneously available and thus a non-negligible influence of the counter-rotating term.

## 3. Few-Cycle-Pulse Adiabatic Floquet Theory

We consider the model (1) written with the time-dependent phase θ+ϕ(t), where the initial phase $\theta \equiv -\phi_{0,i}$ serves for the Floquet representation, and the explicit dependence of the Rabi frequency Ω(t):

(8) $$H^{\Omega (t)}\left(\theta +\phi \left(t\right),t\right)=\frac{\hbar \omega_{0}}{2}\left[\begin{array}{cc} -1 & 0\\ 0 & 1 \end{array}\right]+\hbar \Omega \left(t\right)\cos\left(\theta +\phi \left(t\right)\right)\left[\begin{array}{cc} 0 & 1\\ 1 & 0 \end{array}\right].$$

The quasienergy operator (A19)

(9) $$K\equiv K^{\Omega ,\omega }=-i\hbar \omega \frac{\partial }{\partial \theta }+H^{\Omega }\left(\theta \right)$$

features the two parameters Ω≡Ω(t) and ω≡ω(t) that can be designed independently as functions of time. They will both normalized with $\omega_{0}$. The resonant transformation (6) is represented in the Floquet picture by the transformation (which dresses the upper state with minus one photon):

(10) $$R=\left[\begin{array}{cc} 1 & 0\\ 0 & e^{-i\theta } \end{array}\right],$$

which leads to

(11) $$R^{†}KR=-i\hbar \omega \frac{\partial }{\partial \theta }+\frac{\hbar }{2}\left[\begin{array}{cc} -\Delta (t) & \Omega (t)\\ \Omega (t) & \Delta (t) \end{array}\right]+\frac{\hbar \Omega (t)}{2}\left[\begin{array}{cc} 0 & e^{-2i\theta }\\ e^{2i\theta } & 0 \end{array}\right].$$

Note that we have omitted as before a diagonal term equal to −ω/2 times identity in order to work with traceless matrices (without loss of generality for population transfer consideration).

Adiabatic passage through the (one-photon) resonance, using a pulsed (symmetric) Rabi frequency $\Omega \left(t\right)=\Omega_{0}\Lambda \left(t\right)$ with $\Omega_{0}$ the peak value and the shape 0≤Λ(t)≤1, with Λ(±∞)→0, Λ(0)=1, and of characteristic width τ, and a chirped frequency ω(t), of characteristic width around the resonance $\Delta_{0}$, requires in general $\Omega_{0}∼\Delta_{0}\gg 1/\tau$. The chirped frequency induces for the full coupling Ω(t)cosϕ(t) in Equation (1) the phase $\phi \left(t\right)=\int_{t_{i}}^{t}\omega \left(s\right)ds$, which can be rewritten as a function of the detuning; see Equation (2): $\phi \left(t\right)=\omega_{0}\left(t-t_{i}\right)-\int_{t_{i}}^{t}\Delta \left(s\right)ds-\phi_{0,i}$. We consider smooth Gaussian pulses $\Lambda \left(t\right)=e^{-{\left(t/\tau \right)}^{2}}$, which allows in principle a transfer exponentially accurate as a function of the pulse duration [38] (see Appendix A.2). Defining $\tau \Omega_{0}∼T\Omega_{0}=2\kappa \pi$ (with a larger κ ensuring a better adiabatic passage) leads to $\Omega_{0}/\omega_{0}=\kappa /N$. This shows that adiabatic passage, for a given κ, breaks more strongly the rotating wave approximation for a smaller number N of cycles, since $\Omega_{0}$ gets closer to ω. This requires to take into account the full quasienergy operator.

**Figure 1:**
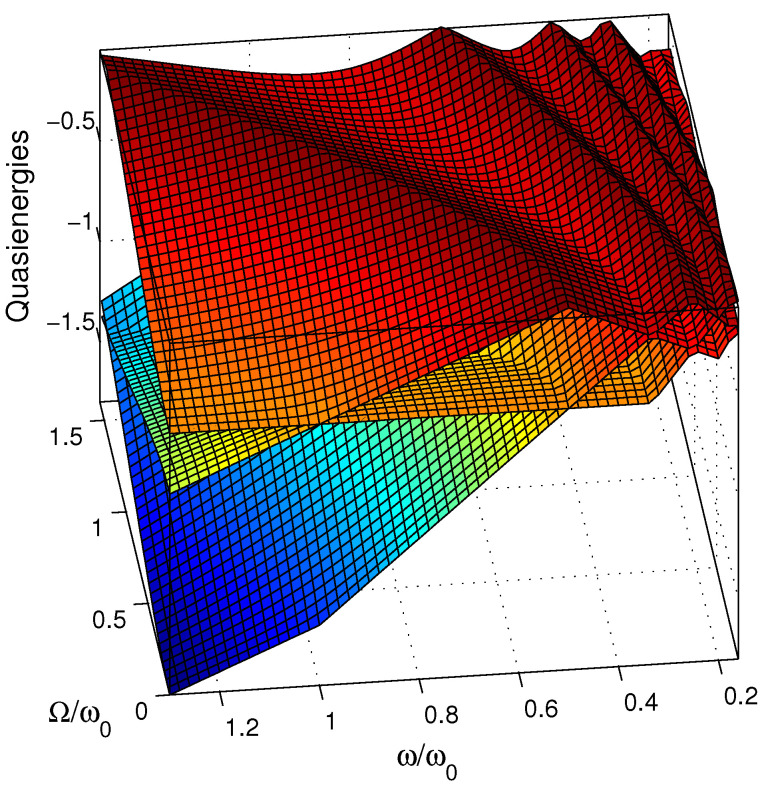
Quasienergy surfaces (in units of $\hbar \omega_{0}$) as functions of $\omega /\omega_{0}$ and $\Omega /\omega_{0}$.

**Figure 2:**
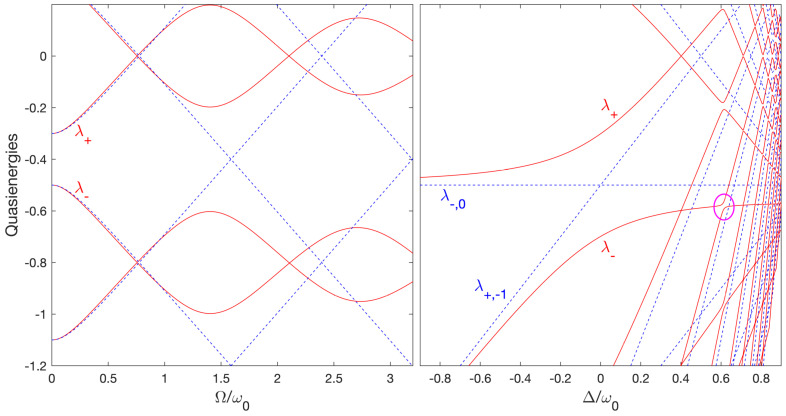
Cross section (full lines) of the quasienergy surfaces (in units of $\hbar \omega_{0}$) of Figure 1 as functions of (i) $\Omega /\omega_{0}$ for a constant $\omega =0.8\omega_{0}$, i.e., $\Delta =0.2\omega_{0}$, where the dashed lines are the quasienergies in the resonant approximation (left frame); (ii) $\Delta /\omega_{0}$ for a constant $\Omega =0.4\omega_{0}$ (full line) and Ω=0 (dashed lines) (right frame). One can distinguish the three-photon resonance as an avoided crossing located around $\Omega =1.4\omega_{0}$ (left frame) and identified by a circle (right frame). The five-photon-resonance avoided crossing can be seen around $\Omega =2.7\omega_{0}$ (left frame).

The quasienergies $\lambda_{\pm ,k}$ can be obtained from the numerical diagonalization of the quasienergy operator (9) [or equivalently (11), apart a change of energy reference], as shown in Figure 1. We emphasize that the quasienergies do not depend on the number of cycles considered N. The complete spectral information is contained in one Floquet zone composed of two surfaces $\lambda_{\pm ,0}$ within a given band of energy of width ℏω. The other surfaces can be constructed using the periodicity of the spectrum: $\lambda_{\pm ,k}\equiv \lambda_{\pm ,0}+k\hbar \omega$, for any (positive or negative) integer *k*. In Figure 1 we display a few surfaces. Figure 2 shows cross-sections, one for a constant ω (left frame) and for constant Ω (right frame). Around the one-photon resonance $\omega \approx \omega_{0}$ (for $\Omega \ll \omega_{0}$), one can recognize the surfaces of Figure 1 of Ref. [17]: For Ω=0, the horizontal line of energy $-0.5\hbar \omega_{0}$ corresponds to the lower state of the system |−,0〉, with the notation |±,k〉 for the bare state |±〉 dressed with *k* photon, i.e., the state |±〉⊗|k〉 with $|k\rangle \equiv e^{ik\theta }$. The crossing line (at $\omega =\omega_{0}$) corresponds to the upper state dressed with minus one photon |+,−1〉. One can distinguish in the plane Ω=0 resonances appearing as *crossings* at $\omega =\omega_{0}/\left(2k+1\right)$ with k=0,1,2⋯ For Ω≠0, they become *avoided crossings*. For $\omega =\omega_{0}/\left(2k\right)$, we have exact crossings for any Ω, due to the particular symmetry of this model (one can see an example for k=1). This means that only odd numbers of photons can be absorbed (or emitted) in such a system. The maxima of the upper surface correspond to crossings (for any Ω), and the valleys to avoided crossings (i.e., to resonances). One can observe that for increasing Ω, the position of the resonances are shifted in the direction of larger ω. This can be interpreted as a Stark shift of the states. This implies that moving along a straight line with $\omega \approx \omega_{0}$ for growing Ω allows one to cross dynamically the three-photon resonance, next the five-photon resonance, and so on, as shown in Figure 2. The three-photon resonance avoided crossing represents thus the first dynamical obstruction that can prevent the control of population transfer by a standard chirped pulse for a large enough Rabi frequency.

It is important to emphasize that the labeling of the surfaces can be only local due to the multiple crossings. This is this property which, when the resonance is dynamically crossed (i.e with a change of sign of the detuning during the dynamics), will allow a population transfer by adiabatic passage of a single Floquet eigenstate that will connect state |−,0〉 at early time with state |+,−1〉 at late time [17]. We denote around the one-photon resonance ($\omega \approx \omega_{0}$) the upper (lower) surface as $\lambda_{+}$ ($\lambda_{-}$), respectively. Near the one-photon resonance, the surface $\lambda_{+}$ ($\lambda_{-}$) is connected to the state |+,−1〉 (|−,0〉) for $\omega <\omega_{0}$, i.e., Δ>0, and to the state |−,0〉 (|+,−1〉) for $\omega >\omega_{0}$, i.e., Δ<0, respectively.

To achieve population transfer, we apply the strategy of parallel adiabatic passage, where the adiabatic dynamics follows a level line $\lambda_{+}-\lambda_{-}=const.$ around the resonance, which is known to be efficient in the context of RWA for a smooth Rabi frequency [39,40,41]. In the RWA, the detuning crosses the resonance according to

(12) $$\Delta \left(t\right)=\pm \sqrt{\Omega_{0}^{2}-\Omega^{2}\left(t\right)},\;\Delta_{0}=\Omega_{0}>0,$$

with the sign + (−) for t<0 (t>0), i.e., $\Delta \left(-\infty \right)=\Delta_{0}$ [$\Delta \left(+\infty \right)=-\Delta_{0}$] the initial (final) detuning, respectively. We remark that, in the RWA, the symmetric situation: the sign − (+) for t<0 (t>0), i.e., $\Delta \left(-\infty \right)=-\Delta_{0}$ [$\Delta \left(+\infty \right)=\Delta_{0}$] the initial (final) detuning, respectively, leads to the same population transfer. As mentioned above, the quality of adiabatic passage can be evaluated by the value of $\tau \Delta_{0}$. It has been shown in [39,41] that Gaussian-pulse parallel adiabatic passage mainly features (i) high fidelity (corresponding to population transfer with a deviation from the perfect transfer of the order $10^{-3}$) for the product $\tau \Delta_{0}$ as low as $\tau \Delta_{0}\approx 2.4$, (ii) a narrow perfect transfer for $\tau \Delta_{0}\approx 2.58$, and (iii) ultrahigh fidelity for $\tau \Delta_{0}≳4.5$.

**Figure 3:**
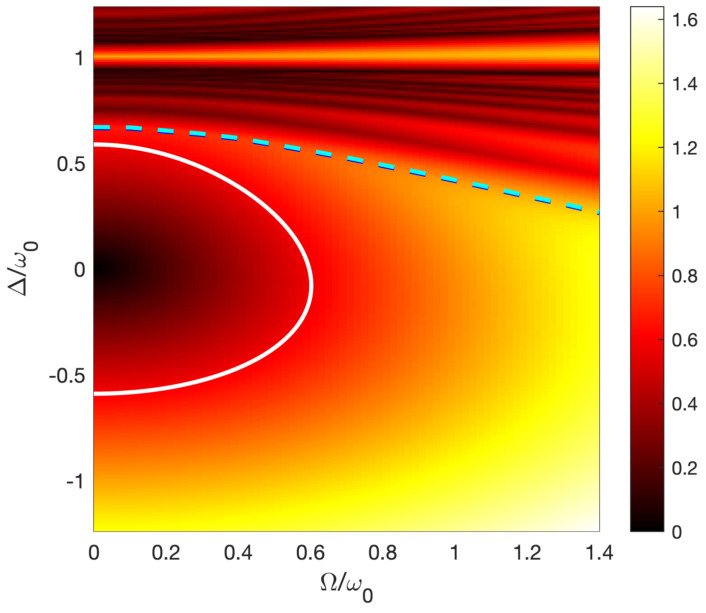
Contour plot of $|\lambda_{+}-\lambda_{-}|$ around the one-photon resonance. The dashed line shows the position of the three-photon resonance, which occurs for a smaller $\Omega /\omega_{0}$ when one considers a larger detuning $\Delta /\omega_{0}$. The level line trajectory (full line) slightly avoids the three-photon-resonance curve (for positive detunings).

In the situation of small number of cycles N, one has additionally to keep a sufficient distance from the neighbouring three-photon resonance. The level lines around the one-photon resonance correspond thus to the constant distances $|\lambda_{+}-\lambda_{-}|$ which are compared to the three-photon resonance, as represented in Figure 3. The driving phase corresponding to a given trajectory of Figure 3, where the pulse amplitude is chosen as a Gaussian pulse of amplitude $\Omega_{0}=\Delta \left(-\infty \right)$, is given by (2). The initial phase $\phi_{0,i}$, which does not modify the dynamics in the adiabatic limit, will be chosen to satisfy the zero time-integrated area (5).

Considering N oscillations in the pulse, i.e., $T\omega_{0}/2\pi =N$, and Gaussian pulses with the estimate T∼4πτ/3 for the pulse duration gives $\tau \omega_{0}\approx 3N/2$, i.e.,

(13) $$\tau \Delta_{0}\approx \frac{3}{2}N\frac{\Delta_{0}}{\omega_{0}}.$$

This shows that smaller values N require larger $\Delta_{0}/\omega_{0}$ to keep the same $\tau \Delta_{0}$.

**Figure 4:**
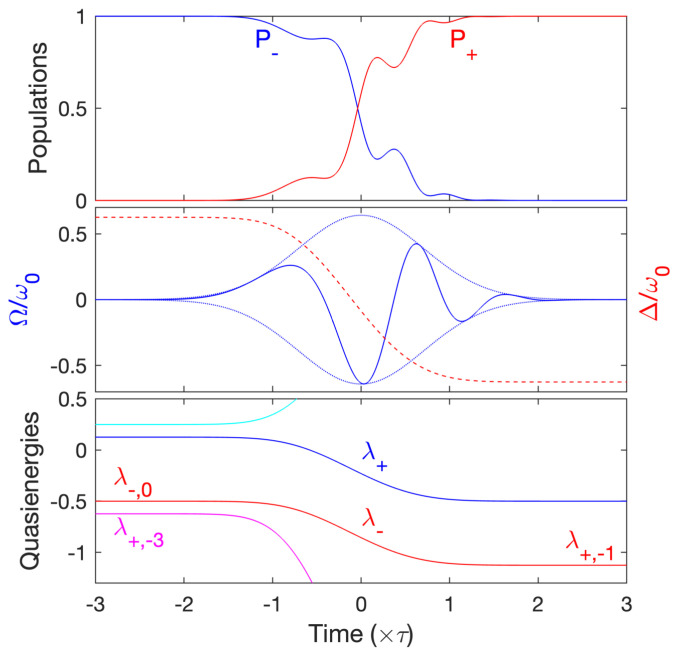
Dynamics along the level line (a) of Figure 3 with $\Delta \left(-\infty \right)=0.625\omega_{0}$ and $\tau \omega_{0}=3.75$ for a Gaussian pulse $\Omega \left(t\right)=\Omega_{0}e^{-{\left(t/\tau \right)}^{2}}$, giving N≈2.5. Upper frame: Populations $P_{j}:={\left|\langle j|\varphi \left(t\right)\rangle \right|}^{2}$. We obtain an ultrahigh fidelity $P_{+}\approx 0.9999$. Middle frame: the normalized Rabi frequency (full line) with its envelope (dotted line) and the instantaneous normalized detuning (dashed line). Lower frame: the instantaneous quasienergies (in units of $\hbar \omega_{0}$). The dynamics follows $\lambda_{-}$ [connected to |−,0〉 (|+,−1〉) at early (late) times], close to the three-photon resonance quasienergy $\lambda_{+,3}$ at early times.

We have obtained the most extreme situation with a low number of cycles N≈2.5 allowing ultrahigh-fidelity adiabatic passage with the following parameters. We consider the trajectory shown in Figure 3 around the one-photon resonance $\Delta_{0}/\omega_{0}=0.625$, which slightly avoids the three-photon-resonance curve, and the smallest quantity $\tau \Delta_{0}=2.35$ satisfying adiabatic passage (see the discussion above). We have determined numerically the value $\phi_{0,i}\approx -0.3032\pi$ satisfying the zero time-integrated area (5). Despite the close presence of the three-photon resonance, we have obtained a remarkable ultrahigh fidelity, as shown in Figure 4. Note that we have chosen a dynamics such that $\Delta \left(-\infty \right)=\Delta_{0}$; we have checked that we have the same final result if we start with $\Delta \left(-\infty \right)=-\Delta_{0}$ (but with a different initial phase $\phi_{0,i}$). We notice that, in practice, since the dynamics does not strictly satisfy the adiabatic conditions, the population dynamics slightly depends on this initial phase.

Decreasing the number of cycles degrades the fidelity because one cannot satisfy a sufficiently large $\tau \Delta_{0}$ from (13).

## 4. Few-Cycle Generalized π-Pulse and Non-Adiabatic Regimes

### 4.1. Definition

In this section and the next ones, one studies few-cycle pulses driving the dynamics in nonadiabatic regimes and analyzes how one can control the population transfer, in particular with ultrahigh fidelity.

We introduce the notion of few-cycle generalized π-pulse by expanding the Rabi frequency as a Fourier series:

(14) $$\Omega \left(t\right)=\sum_{n\geq 1}\Omega_{n}\left(t\right),\;\mathrm{with}\;\Omega_{n}\left(t\right)=\Omega_{n}\Lambda_{n}\left(t\right)\;\mathrm{and}\;\Omega_{n}\geq 0,$$

with the envelope of each pulsed mode defined as

(15) $$\Lambda_{n}\left(t\right)=\frac{1}{2}\left[1-{\left(-1\right)}^{n}\cos\left(\frac{n\omega_{0}}{N}t\right)\right],$$

which satisfies $0\leq \Lambda_{n}\left(t\right)\leq 1$. This particular Fourier expansion (14) implies that Ω(t)≥0 and $\Omega \left(t_{i}\right)=\Omega \left(t_{f}\right)=0$. Moreover, one can prove that, for $\phi \left(t\right)=\omega_{0}\left(t-t_{i}\right)-\phi_{0,i}$ (i.e., exact resonance, Δ=0, and for any initial phase), imposing $\Omega_{N}=0$ guarantees that the zero area condition (5) is satisfied.

If one considers a single field $\Omega \left(t\right)\equiv \Omega_{1}\left(t\right)$, the high-frequency effective Hamiltonian from the counter-rotating term (11) reads [see Equation (A52)], where we have neglected the terms of Equation (A55) induced by the time-dependence of $\Omega_{1}\left(t\right)$:

(16) $$\tilde{H}\approx \frac{1}{2}\left[\begin{array}{cc} -\left(\frac{\Omega_{1}^{2}\left(t\right)}{4\omega_{0}}+\Delta \left(t\right)\right) & \Omega_{1}\left(t\right)\\ \Omega_{1}\left(t\right) & \frac{\Omega_{1}^{2}\left(t\right)}{4\omega_{0}}+\Delta \left(t\right) \end{array}\right].$$

The diagonal term can be neglected with respect to the coupling when (considering the condition for the peak Rabi frequencies) $\Omega_{1}\gg \frac{1}{2}\frac{\Omega_{1}^{2}}{\omega_{0}}.$ In the π-pulse regime, the pulse area of the pulse envelope is then $\Omega_{1}T/2=\pi$, and the above condition becomes

(17) N≫1/2

to neglect the diagonal term. This consists precisely to one of the RWA conditions, i.e., a large number of resonant cycles.

### 4.2. Few-Cycle Resonant Rabi Oscillations

**Figure 5:**
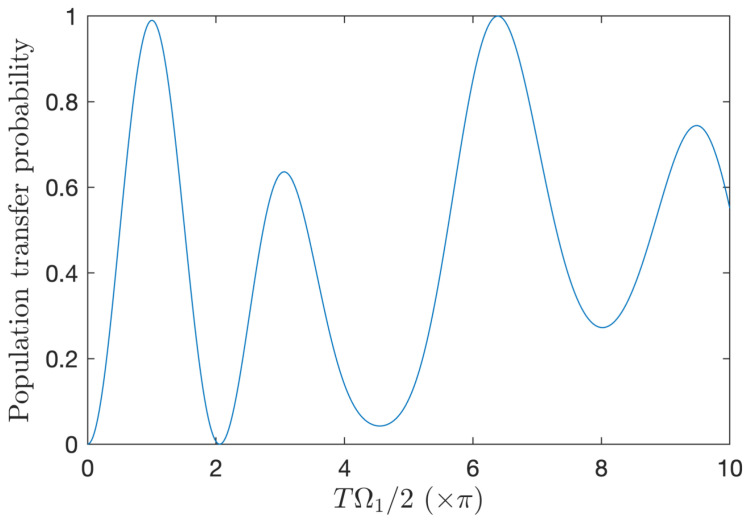
Two-cycle resonant Rabi oscillations: Final population transfer probability to the upper state as a function of the pulse area (×π) for a single pulse $\Omega_{1}\left(t\right)$ and the number of cycles N=2 using the full model (1). The transfer reaches a local maximum ($P_{+}\approx 0.99$) close to the standard π-pulse condition but does not reach ultrahigh fidelity (except for an undesirable large pulse area $T\Omega_{1}/2\approx 6.38\pi$).

**Figure 6:**
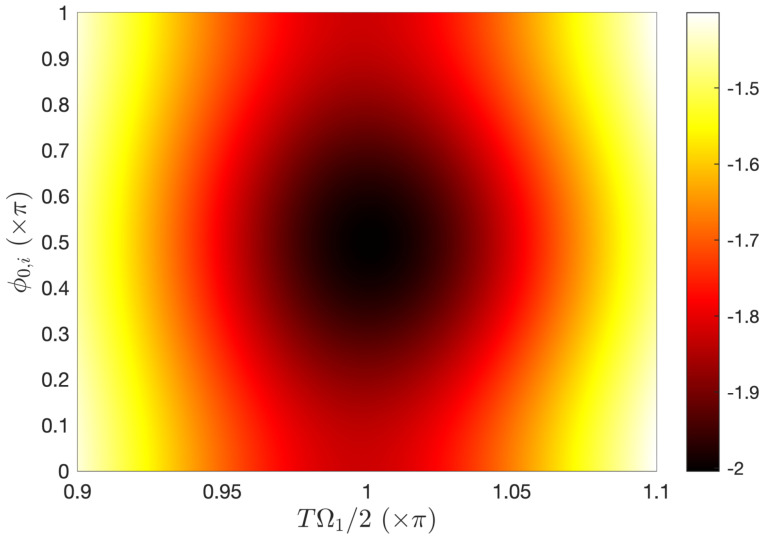
Contour plot of $\log_{10}\left(1-P_{+}\right)$ with $P_{+}$ the final population transfer probability around the π-pulse regime in the condition of Figure 5, but with varying initial phases $\phi_{0,i}$.

In the resonant situation, Δ=0, we consider the number of cycles N=2 and $\Omega_{2}=0$ such that the zero area condition (5) is satisfied. Figure 5 shows the few-cycle Rabi oscillations produced for the phase of the field $\phi \left(t\right)=\omega_{0}\left(t-t_{i}\right)\pm \pi /2$ (i.e., $\phi_{0,i}=\mp \pi /2$) leading to the maximum population transfer (see Figure 6 and the discussion below). They globally significantly deviate from the standard RWA Rabi oscillations; however, they feature a rather good maximum final transfer, $P_{+}\approx 0.99$ for a peak amplitude $\Omega_{1}\approx 2\pi /T=\omega_{0}/N$ close to the standard π-pulse condition (with a relative deviation of order $2\times 10^{-3}$). It also shows an ultrahigh-fidelity population transfer but for a high and quite unrealistic pulse area $T\Omega_{1}/2\approx 6.38\pi$. Figure 6 shows the weak dependance of the population transfer over the initial phase $\phi_{0,i}$ near the π-pulse regime. The maximum is obtained for the initial phase $\phi_{0,i}=\pm \pi /2$. We have determined that the population transfer depends on $\phi_{0,i}$ much more strongly for higher pulse area regimes (not shown).

**Figure 7:**
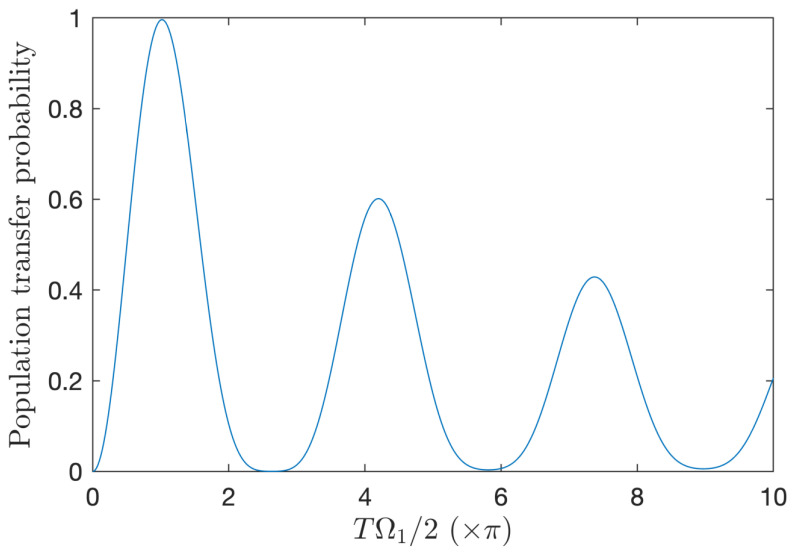
Single-cycle resonant Rabi oscillations: Same as Figure 5 but for a single cycle N=1. The transfer reaches a good fidelity $P_{+}\approx 0.996$ close to the standard π-pulse condition: $\Omega_{1}T/2\approx 1.018\pi$.

Figure 7 shows the single-cycle Rabi oscillations, N=1, for the phase of the field $\phi \left(t\right)=\omega_{0}\left(t-t_{i}\right)\pm \pi /2$, which satisfies the zero time-integrated area condition (5). Surprisingly, the maximum transfer (which occurs close to the standard π-pulse condition) is even better in this case compared to N=2 (for the same pulse area), while the system deviates more from the RWA according to condition (17). This can be attributed to the effect of significant population transfer by off-resonant zero-area pulse described in [42], which counterbalances the deviation from RWA. We notice that this effect does not apply efficiently when the field amplitude increase due to the form of the interaction, which is not fully compatible with the hypothesis of [42] in the limit of strong field (as it does not tend to a Dirac δ distribution type interaction).

However, in both situations, N=1,2, the transfer is not perfect, and cannot reach ultrahigh fidelity near the π-pulse regime.

Our goal in the following consists in determining strategies of pulse shaping inducing ultrahigh fidelity in a π-pulse regime. We will show that this will be achieved by adding higher-order terms $\Omega_{n}$, that will allow us to alleviate condition (17), or by considering a chirped frequency (next Section). A more systematic optimization procedure will be conducted in Section 6.

### 4.3. 2N-Resonance Strategy

We consider here the situation N≥2 and $\phi \left(t\right)=\omega_{0}t$, i.e., an exactly resonant problem Δ=0, with $\phi_{0,i}=-\omega_{0}t_{i}$. We add a single 2N-mode to the main mode: $\Omega \left(t\right)=\Omega_{1}\left(t\right)+\Omega_{2N}\left(t\right)$. The zero area condition (5) is automatically satisfied since $\Omega_{N}=0$. In this case, the fast oscillating n=2N-mode term features a resonance with the counter-rotating coupling:

$$\begin{array}{l} \tilde{H}=\frac{1}{2}\left(\Omega_{1}\left(t\right)+\frac{1}{4}\Omega_{2N}\right)\left[\begin{array}{cc} 0 & 1\\ 1 & 0 \end{array}\right]\\ +\frac{1}{2}\left(\Omega_{1}\left(t\right)\left[\begin{array}{cc} 0 & e^{-2i\omega_{0}t}\\ e^{2i\omega_{0}t} & 0 \end{array}\right]+\frac{1}{4}\Omega_{2N}\left[\begin{array}{cc} 0 & e^{-2i\omega_{0}t}\\ e^{2i\omega_{0}t} & 0 \end{array}\right]-\frac{1}{4}\Omega_{2N}\left[\begin{array}{cc} 0 & e^{2i\omega_{0}t}\\ e^{-2i\omega_{0}t} & 0 \end{array}\right]\right) \end{array}$$

(18) $$\;-\frac{1}{8}\Omega_{2N}\left[\begin{array}{cc} 0 & e^{-4i\omega_{0}t}\\ e^{4i\omega_{0}t} & 0 \end{array}\right].$$

If we neglect the oscillating terms [second and third lines of (18)], the area of the resulting coupling is

(19) $$\begin{array}{c} \int_{-T/2}^{T/2}\left(\Omega_{1}\left(t\right)+\frac{1}{4}\Omega_{2N}\right)dt=\frac{T}{2}\left(\Omega_{1}+\frac{1}{2}\Omega_{2N}\right), \end{array}$$

which should be π at the lowest order:

(20) $$\begin{array}{c} \Omega_{1}+\frac{1}{2}\Omega_{2N}=\frac{\omega_{0}}{N}. \end{array}$$

According to (A52) and neglecting the faster oscillating term, one concludes that the dominant correction is given by the $\Omega_{1}\left(t\right)$ counter-rotating term [first term of the second line of (18)]. Considering the peak terms, one can evaluate the (diagonal) correction:

(21) $$\begin{array}{c} {\tilde{H}}_{\max}=\frac{1}{2}\left(\Omega_{1}+\frac{1}{4}\Omega_{2N}\right)\left[\begin{array}{cc} 0 & 1\\ 1 & 0 \end{array}\right]+\frac{\Omega_{1}^{2}}{8\omega_{0}}\left[\begin{array}{cc} -1 & 0\\ 0 & 1 \end{array}\right]. \end{array}$$

Its difference should be much smaller than the coupling term, giving the condition of the ratio

(22) $$\begin{array}{c} R=\frac{\Omega_{1}^{2}}{2\omega_{0}\left(\Omega_{1}+\frac{1}{4}\Omega_{2N}\right)}=\frac{\Omega_{1}^{2}}{\omega_{0}(\Omega_{1}+\frac{\omega_{0}}{N})}\ll 1. \end{array}$$

We implemented a systematic search over the parameter $\Omega_{1}$ for N=2 and found an ultrahigh-fidelity transfer $P_{+}\approx 0.9999$ for $\Omega_{1}\approx 0.276\omega_{0}$ [leading to $\Omega_{4}\approx 0.448\omega_{0}$ according to (20)], corresponding to an equal distribution of both contributions in (20): $\Omega_{1}∼\frac{1}{2}\Omega_{2N}∼\omega_{0}/\left(2N\right)$. This gives in this situation for the above condition

(23) $$\begin{array}{c} N\gg 1/6, \end{array}$$

which significantly alleviates condition (17) (by a factor of 3).

### 4.4. Two-Modes-Resonance Strategy

In this strategy, still with N≥2, we consider alternatively two additional nonresonant fields $\Omega_{2N-1}$ and $\Omega_{2N+1}$ (and $\phi \left(t\right)=\omega_{0}t$, i.e., Δ(t)=0) with the simplifying condition $\Omega_{2N-1}=\Omega_{2N+1}\equiv \Omega_{0}$, giving the total field

(24) $$\begin{array}{c} \Omega \left(t\right)=\frac{\Omega_{1}}{2}\left[1+\cos\left(\frac{\omega_{0}}{N}t\right)\right]+\Omega_{0}\left[1+\cos\left(\frac{\omega_{0}}{N}t\right)\cos\left(2\omega_{0}t\right)\right]. \end{array}$$

This field satisfies the zero-area condition (5) as before since $\Omega_{N}=0$. The Hamiltonian shows that the two fields generate a resonance:

$$\begin{array}{l} \tilde{H}=\frac{1}{2}\left[\Omega_{1}\left(t\right)+\Omega_{0}+\frac{1}{2}\Omega_{0}\cos\left(\frac{\omega_{0}}{N}t\right)\right]\left[\begin{array}{cc} 0 & 1\\ 1 & 0 \end{array}\right]\\ +\frac{1}{4}\Omega_{0}\cos\left(\frac{\omega_{0}}{N}t\right)\left(\left[\begin{array}{cc} 0 & e^{2i\omega_{0}t}\\ e^{2i\omega_{0}t} & 0 \end{array}\right]+\left[\begin{array}{cc} 0 & e^{-2i\omega_{0}t}\\ e^{-2i\omega_{0}t} & 0 \end{array}\right]\right) \end{array}$$

(25) $$\;+\frac{1}{2}\left[\Omega_{1}\left(t\right)+\Omega_{0}\right]\left[\begin{array}{cc} 0 & e^{-2i\omega_{0}t}\\ e^{2i\omega_{0}t} & 0 \end{array}\right]+\frac{1}{4}\Omega_{0}\cos\left(\frac{\omega_{0}}{N}t\right)\left[\begin{array}{cc} 0 & e^{-4i\omega_{0}t}\\ e^{4i\omega_{0}t} & 0 \end{array}\right].$$

The area of the resulting coupling, which should be π, is

(26) $$\begin{array}{c} \int_{-T/2}^{T/2}\Omega \left(t\right)dt=T\left(\frac{\Omega_{1}}{2}+\Omega_{0}\right)=\pi . \end{array}$$

Neglecting the fastest oscillating term, we obtain for the effective Hamiltonian

(27) $$\begin{array}{cc} \; & \tilde{H}\approx \frac{1}{2}\left[\Omega_{1}\left(t\right)+\Omega_{0}+\frac{1}{2}\Omega_{0}\cos\left(\frac{\omega_{0}}{N}t\right)\right]\left[\begin{array}{cc} 0 & 1\\ 1 & 0 \end{array}\right]+\frac{1}{8\omega_{0}}{\left[\Omega_{1}\left(t\right)+\Omega_{0}\right]}^{2}\left[\begin{array}{cc} -1 & 0\\ 0 & 1 \end{array}\right], \end{array}$$

i.e., for the peak values:

(28) $$\begin{array}{cc} \; & {\tilde{H}}_{\max}\approx \frac{1}{2}\left(\Omega_{1}+\frac{3}{2}\Omega_{0}\right)\left[\begin{array}{cc} 0 & 1\\ 1 & 0 \end{array}\right]+\frac{{\left(\Omega_{1}+\Omega_{0}\right)}^{2}}{8\omega_{0}}\left[\begin{array}{cc} -1 & 0\\ 0 & 1 \end{array}\right]. \end{array}$$

The ratio of the (peak) diagonal correction with respect to the (peak) coupling is given by

(29) $$\begin{array}{c} R=\frac{{\left(\Omega_{1}+\Omega_{0}\right)}^{2}}{2\omega_{0}\left(\Omega_{1}+\frac{3}{2}\Omega_{0}\right)}\ll 1. \end{array}$$

This ratio is minimum when $\Omega_{1}=0$: $R=\Omega_{0}/\left(3\omega_{0}\right)=\pi /\left(3T\omega_{0}\right)$. On the contrary, when $\Omega_{0}=0$, the ratio is maximum. This solution minimizing the correction for the complete transfer is thus for $\Omega_{1}=0$ and $T\Omega_{0}=\pi$, i.e.,

(30) $$\begin{array}{c} \Omega_{0}=\frac{\omega_{0}}{2N}. \end{array}$$

The diagonal terms can be neglected, from the above condition, when

(31) N≫1/6.

This shows that the introduction of the two fields in the limit of weak $\Omega_{1}$ allows one to alleviate again condition (17) by a factor of 3, similarly to the preceding strategy.

## 5. Chirped Few-Cycle Pulses: Stark-Shift Compensation Strategy

The effective Hamiltonian (16) with a single-mode field $\Omega \left(t\right)=\Omega_{1}\left(t\right)$ indicates that the lowest order perturbative correction induces a dynamical (diagonal) Stark shift. It can be compensated by a detuning properly shaped [according to (A55) if one considers additionally the time dependence of $\Omega_{1}\left(t\right)$]:

(32) $$\Delta \left(t\right)=-\frac{\Omega_{1}^{2}\left(t\right)}{4\omega_{0}}+\frac{\Omega_{1}^{2}}{64N^{2}\omega_{0}}\sin^{2}\left(\frac{\omega_{0}}{N}t\right)\approx -\frac{\omega_{0}}{32N^{2}}\left[3+4\cos\left(\frac{\omega_{0}}{N}t\right)+\cos\left(\frac{2\omega_{0}}{N}t\right)\right],$$

still with a π-pulse: $\Omega_{1}T/2=\pi$, where we have neglected the second term which is much smaller than the first one. The phase (2) can be then written

$$\begin{array}{cc} \phi (t)= & \left(1+\frac{3}{32N^{2}}\right)\omega_{0}\left(t-t_{i}\right)+\frac{1}{8N}\left[\sin\left(\frac{\omega_{0}}{N}t\right)-\sin\left(\frac{\omega_{0}}{N}t_{i}\right)\right] \end{array}$$

(33) $$\;+\frac{1}{64N}\left[\sin\left(\frac{2\omega_{0}}{N}t\right)-\sin\left(\frac{2\omega_{0}}{N}t_{i}\right)\right]-\phi_{0,i},$$

where the initial phase $\phi_{0,i}$ has to be chosen to satisfy the zero-area condition (5). We have checked that the dynamics weakly depends on its precise value (with a final population transfer oscillating between 0.9996 and 0.9998 as a function of the initial phase). One can prove that the constant part of the phase has to be ±π/2 in order to satisfy (5) in this case of a single-mode field. The phase takes then the form (where the constant part of the phase +π/2 has been chosen)

(34) $$\begin{array}{c} \phi \left(t\right)=\frac{\pi }{2}+{\tilde{\omega }}_{0}t-{\tilde{\phi }}_{0}\left(t\right) \end{array}$$

with, respectively, ${\tilde{\omega }}_{0}$ the mean frequency, which is the frequency that has to be tuned in practice, and ${\tilde{\phi }}_{0}\left(t\right)$ the modulated phase which is the part of the phase that has to be shaped:

(35) $$\begin{array}{c} {\tilde{\omega }}_{0}=\left(1+\frac{3}{32N^{2}}\right)\omega_{0},\;{\tilde{\phi }}_{0}\left(t\right)=-\frac{1}{8N}\sin\left(\frac{\omega_{0}}{N}t\right)\left[1+\frac{1}{4}\cos\left(\frac{\omega_{0}}{N}t\right)\right]. \end{array}$$

**Figure 8:**
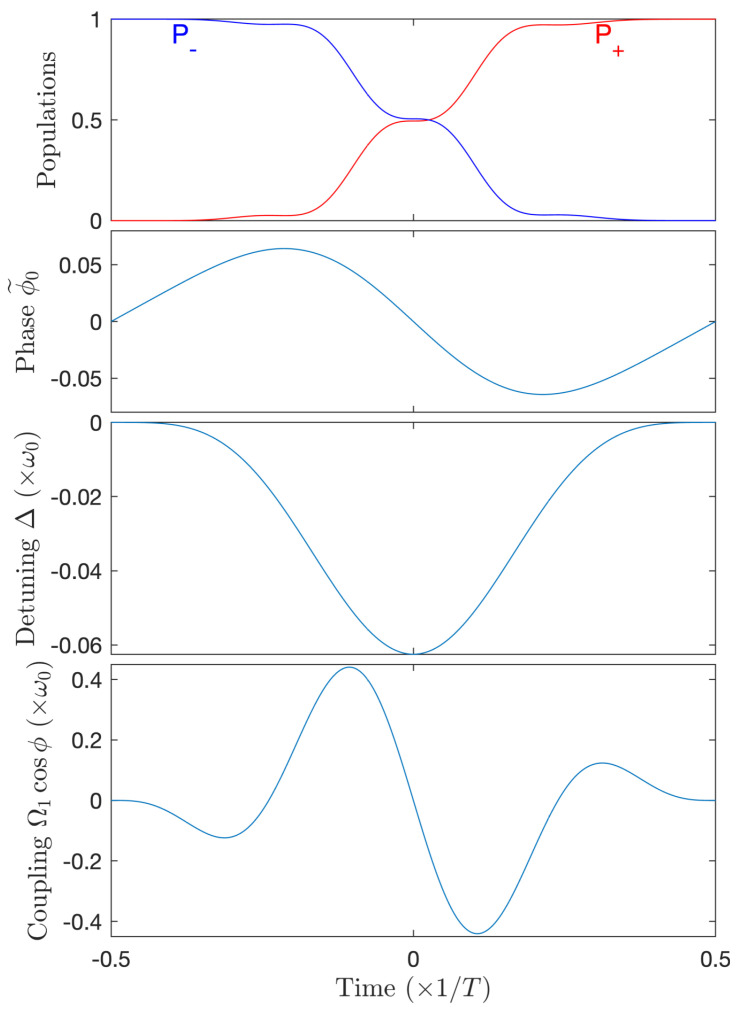
High-fidelity population transfer for N=2 with a single π-pulse mode $\Omega \left(t\right)=\Omega_{1}\left(t\right)$ and the phase (34) featuring a chirped frequency. From top to bottom frames: population dynamics, modulated phase ${\tilde{\phi }}_{0}\left(t\right)$, detuning Δ(t), and full coupling $\Omega_{1}\left(t\right)\cos\phi \left(t\right)$.

Equation (35) shows a small shift of the mean frequency compared to the resonance $\omega_{0}$ and a small phase modulation amplitude (of approximately 1/8N). For instance, we obtain a mean frequency ${\tilde{\omega }}_{0}=1.0234\omega_{0}$ and an approximate phase modulation amplitude of 0.0625 for N=2. Figure 8 shows the phase shaping and the dynamics in this case resulting into the transfer $P_{+}\approx 0.9997$, close to an ultrahigh-fidelity transfer (and more than 30 times more accurate than the optimal nonchirped pulse shown in Figure 5).

More generally, we can show that the phase can take the following form

(36) $$\phi \left(t\right)=\frac{\pi }{2}+{\tilde{\omega }}_{0}t-\sum_{n\geq 1}{\tilde{\phi }}_{0,n}\sin\left(\frac{n\omega_{0}}{N}t\right),$$

i.e., π/2 added to an odd function featuring modulating modes, with ${\tilde{\omega }}_{0}$ the mean frequency, in order to satisfy condition (5) with an even single-mode field $\Omega \left(t\right)=\Omega_{1}\left(t\right)$, for any integer number of cycles N. This will be used for a more systematic optimization in the next section.

## 6. Numerical Optimization

**Figure A1:**
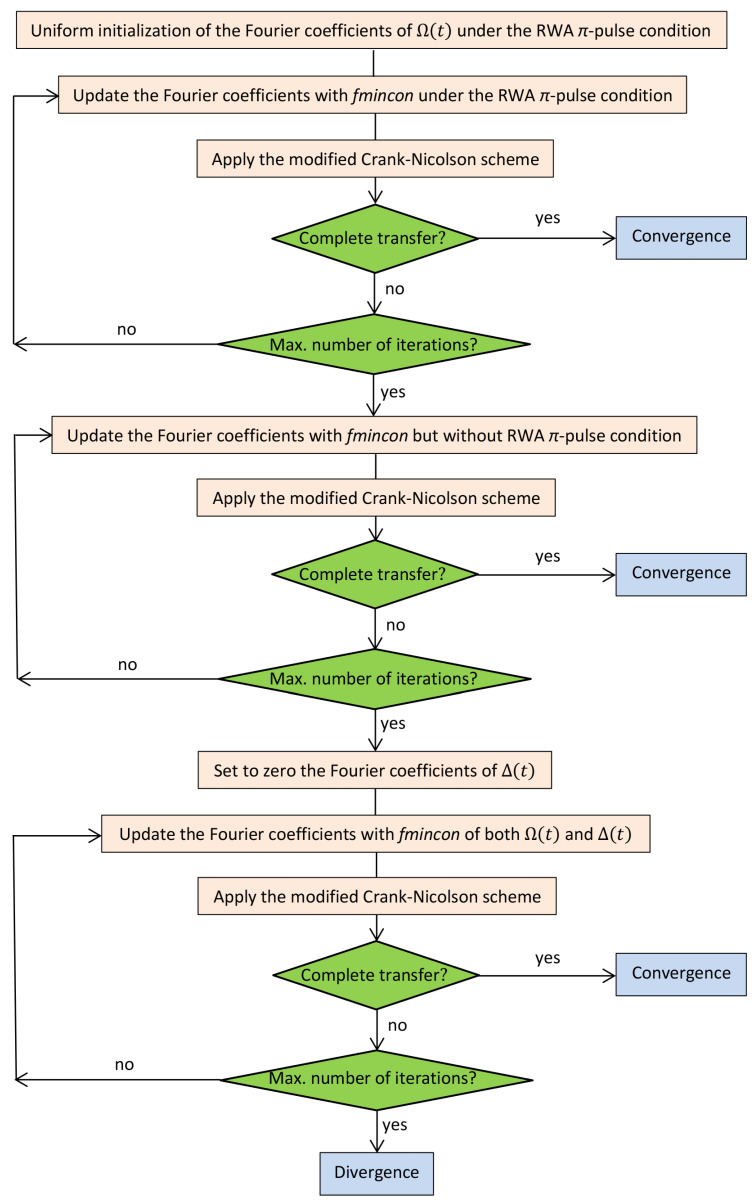
Flowchart of the optimization algorithm. The modified Crank-Nicolson scheme can be found in Ref. [44].

We provide the numerical results of a more systematic optimization procedure for the population transfer problem as described in Appendix B (see the flowchart of Figure A1). Since the parameter landscape is *a priori* large, we orient the search around the three strategies investigated in Section 4 and Section 5 and check the convergence of our algorithm in each situation. We consider the number of cycles N=2 for the strategies where the detuning is zero, which necessitates the cancellation of the n=N mode. On the other hand, we investigate the single-cycle limit N=1 for the more flexible situation with a time-dependent detuning (i.e., a chirped frequency).

### 6.1. 2N-Resonance Strategy

**Figure 9:**
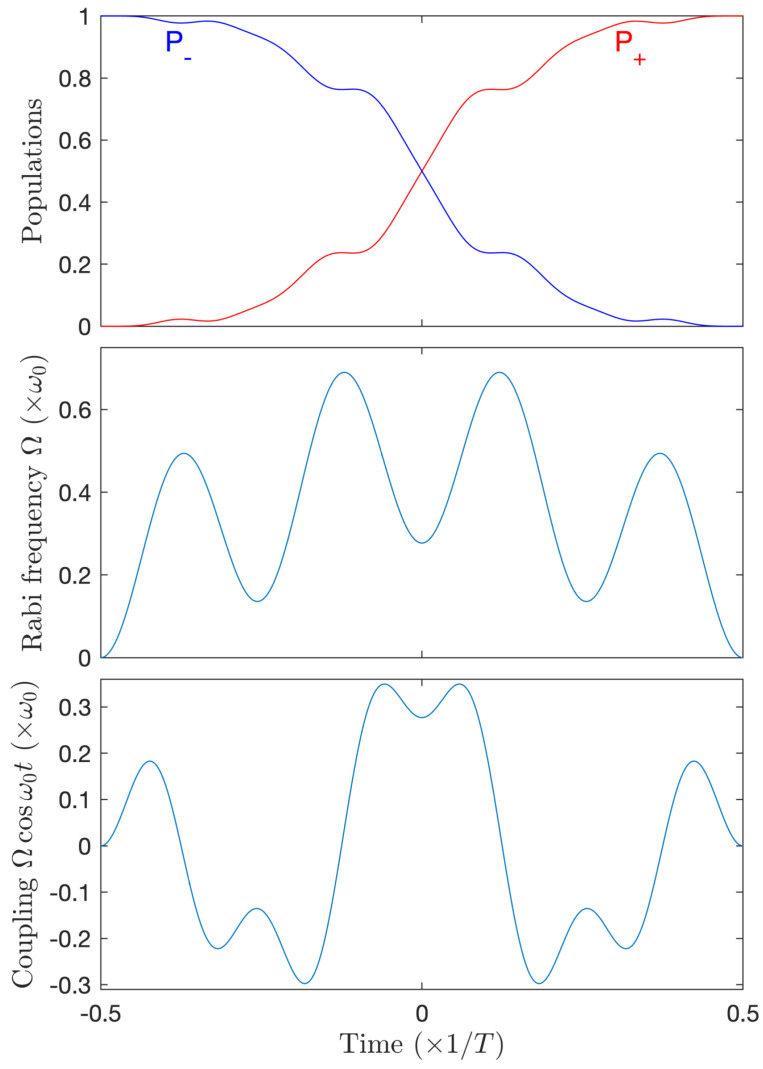
Complete population transfer for N=2 and a four-mode Fourier decomposition with the nonzero modes $\Omega_{1}=0.277\omega_{0},\Omega_{4}=0.452\omega_{0}$ (2N-resonance strategy) resulting from the optimization procedure. Upper frame: population dynamics. Middle frame: pulse envelope Ω(t). Lower frame: Full coupling $\Omega \left(t\right)\cos\omega_{0}t$.

In order to target and validate this strategy, we impose the resonance, $\phi \left(t\right)=\omega_{0}t$, and we implement an optimization using an expansion of Ω(t) limited to the first 4=2N modes for N=2. The mode n=2 is set to zero in order to satisfy Equation (5). Note that, without imposing it, the optimization procedure leads to the vanishing of the third mode n=3, as predicted in Section 4.3. With this strategy, one obtains a complete transfer for $\Omega_{1}\approx 0.277\omega_{0}$ and $\Omega_{4}\approx 0.452\omega_{0}$, see Figure 9. These parameters are close to the ones determined in Section 4.3. The area of the full envelope Ω(t) gives 1.5π. The area $\Omega_{1}\left(t\right)+\frac{1}{4}\Omega_{2N}$ is, as predicted, close to π: $T(\Omega_{1}+\frac{1}{2}\Omega_{4})\approx 1.006\pi$.

Adding more modes, as proposed in the next subsection, will allow one to explore a different strategy to achieve the same complete transfer result.

### 6.2. Two-Modes-Resonance Strategy

**Figure 10:**
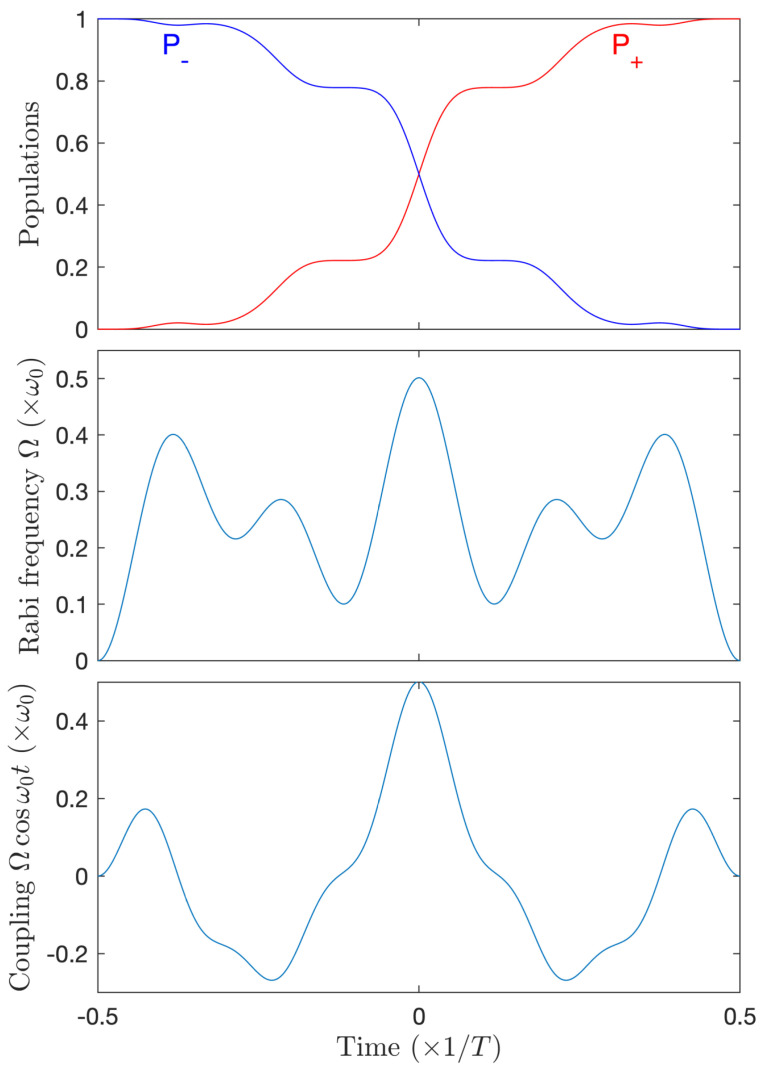
Same as Figure 9 but for a five modes decomposition with the obtained non-zero modes $\Omega_{1}=0.043\omega_{0},\Omega_{3}=0.2314\omega_{0},\Omega_{5}=0.227\omega_{0}$ (two-modes-resonance strategy).

We now consider the resonant case $\phi \left(t\right)=\omega_{0}t$ with an expansion of Ω over the first 5=2N+1 modes still for N=2. Adding the fifth mode opens a path to the two-modes-resonance strategy described in Section 4.4. The mode 2 is still set to zero in order to satisfy Equation (5) and, without imposing it, the optimization procedure leads to the vanishing of the fourth mode n=4. One also obtains in this case a complete population transfer resulting from the combination of the 2N−1=3 and 2N+1=5 modes. We notice that, as predicted in Section 4.4, the optimized amplitudes $\Omega_{3}$ and $\Omega_{5}$ are close to each other (see Figure 10), with a value consistent with Equation (30).

### 6.3. Stark-Shift Compensation Strategy

One finally considers the situation with a nonzero detuning and a low number of cycles, N=2 or N=1. In both cases, it is sufficient to restrict Ω to the first Fourier mode, $\Omega \left(t\right)=\Omega_{1}\left(t\right)$, and to decompose the phase ϕ(t) according to Equation (36) with a single mode:

(37) $$\begin{array}{c} \phi \left(t\right)=\frac{\pi }{2}+{\tilde{\omega }}_{0}t-{\tilde{\phi }}_{0,1}\sin\left(\frac{\omega_{0}}{N}t\right). \end{array}$$

We obtain in both cases a complete transfer with smooth and simple phase and detuning.

For N=2, the optimized parameters are $\Omega_{1}=0.504\omega_{0}$, ${\tilde{\omega }}_{0}=1.038\omega_{0}$, and ${\tilde{\phi }}_{0,1}=-9.74\times 10^{-3}$, giving a pulse area 1.0087π. The phase can be compared to (34) and (35): It features a larger shift of the mean frequency (though small compared to $\omega_{0}$) and a smaller modulation amplitude (given by the absolute value of ${\tilde{\phi }}_{0,1}$, which is small compared to the angle π), of frequency $\omega_{0}/2$. The resulting dynamics (not shown) is similar to the one obtained in Figure 8.

**Figure 11:**
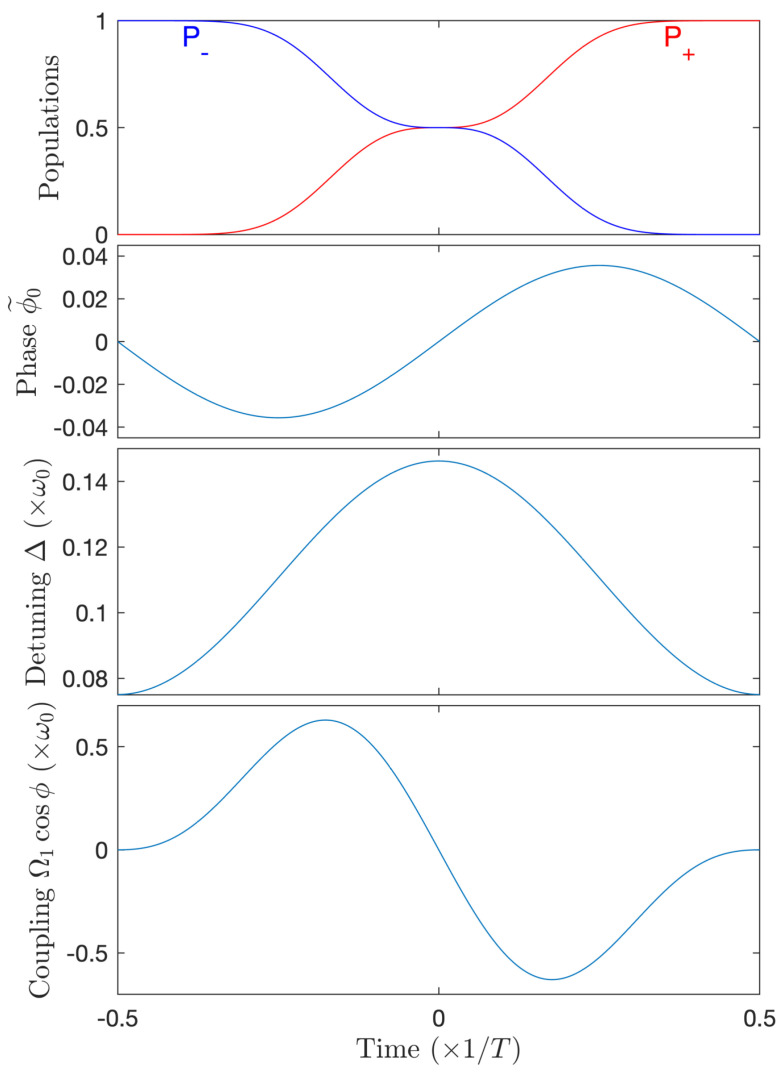
Complete population transfer for N=1, a single-mode for the amplitude: $\Omega \left(t\right)=\Omega_{1}\left(t\right)$ and the phase: $\phi \left(t\right)=\pi /2+{\tilde{\omega }}_{0}t-{\tilde{\phi }}_{0,1}\sin\left(\omega_{0}t\right)$ with the non-zero coefficients $\Omega_{1}=1.067\omega_{0}$, ${\tilde{\omega }}_{0}=0.889\omega_{0}$, ${\tilde{\phi }}_{0,1}=3.56\times 10^{-2}$. From top to bottom frames: population dynamics, modulated phase ${\tilde{\phi }}_{0}\left(t\right)$, detuning Δ(t), full coupling $\Omega_{1}\left(t\right)\cos\phi \left(t\right)$.

Optimization for the case N=1 is displayed in Figure 11. It gives a pulse area 1.067π. One can notice a stronger (and negative, i.e., $\dot{\phi }<\omega_{0}$) shift of the mean frequency (still much smaller than $\omega_{0}$) and a larger modulation amplitude compared to the above case N=2 (but still smaller than the compensation shown in Figure 8), which is still small (compared to the angle π). The modulation frequency of the phase is here $\omega_{0}$.

In both cases, N=1,2, the resulting phase is smooth, it features small deviations and small modulation amplitude at the frequency close to the resonant frequency $\omega_{0}$ and leads to an instantaneous frequency which is also close to $\omega_{0}$. All these ingredients indicate a realistic experimental implementation in principle.

## 7. Conclusions

In this paper, we have explored quantum control with a few-cycle pulse implementing and generalizing two standard strategies, adiabatic Floquet theory and π-pulse. We have shown that adiabatic Floquet is obstructed by the presence of avoided crossing in the quasi-energy spectrum induced by the counter-rotating term for the number of cycles N<2.5. This shows the relevance of using the tool of adiabatic Floquet theory for analyzing and controlling the dynamics even for few cycles.

We have implemented a generalization of π-pulse transfer by expanding the pulse shape in a Fourier expansion. We have shown two particular strategies on resonance with a few modes involved, named 2N-resonance and two-modes-resonance strategies. Complete transfer can be achieved in these cases for a number of cycles as low as N=2. We have also explored a chirped frequency associated to a simple pulsed-shape field. We have shown that an appropriate chirping allows an ultrahigh-fidelity transfer for a number of cycles as low as N=1 (single-cycle pulse).

In a future work, we will explore the control in a multilevel system since the broadening of the spectrum induced by the small number of cycles is expected to impact significantly excited states. The present study will serve as an important exploratory work that will guide the strategies with the clear advantage of limiting the parameters landscape compared to blind optimal control strategies. More specifically, we will use the few-cycle-pulse adiabatic Floquet theory, including multistate effects, such as rotational and vibrational states in molecules (similarly to Ref. [43]), but beyond the RWA (similarly to Figure 1),which will allow one to find adiabatic trajectories as functions of the phase and pulse amplitudes (when they exist), connecting the initial and targeted states. In addition, we have constructed specific parametrizations in amplitude (14) and (15) and in phase (36) that satisfy the physical constraint of zero time-integrated field area (5) with few parameters to be optimized. Nonadiabatic few-cycle regimes with shaped and chirped pulses in multilevel systems will be then explored on this basis. This will find for instance applications in the fine control of angular wavepackets towards the control of the orientation and more generally of the rotation of molecules [15] by single-cycle THz pulses. 

## Appendix A. Adiabatic Floquet Theory

### Appendix A.1. Floquet Theory for Periodic Systems

The Floquet theory allows the treatment of a periodic time dependent system by transforming it into a time-independent problem via the use of an enlarged space as follows [18]. A periodic system of Hamiltonian H(θ+ϕ(t)+2π)=H(θ+ϕ(t)) features a 2π periodic phase, $\phi \left(t\right)=\omega \left(t-t_{i}\right)$ with θ the initial phase at time $t_{i}$, ω the angular frequency. A typical example is

(A1) $$H\left(x;\theta +\omega \left(t-t_{i}\right)\right)=H_{0}\left(x\right)-\mu \left(x\right)E\cos\left(\theta +\omega \left(t-t_{i}\right)\right)$$

for a quantum system of Hamiltonian $H_{0}\left(x\right)$ driven by a periodic field of amplitude E via the dipole moment μ(x). The Schrödinger equation

(A2) $$i\hbar \frac{\partial }{\partial t}\varphi =H\left(x;\theta +\omega (t-t_{i})\right)\varphi ,\;\varphi \in H$$

is defined on a Hilbert space H, which can be of infinite dimension (e.g., the space of square-integrable functions $H=L_{2}\left(R^{n},\;d^{n}x\right)$, where *n* is the number of the degrees of freedom of the molecule) or of finite dimension (e.g., in *N*-level models $H=C^{N}$). The initial phase θ appears as a parameter. One can think of Equation (A2) as a family of equations parameterized by the angle θ. We define the solution ψ(t) of a time independent problem:

(A3) $$i\hbar \frac{\partial }{\partial t}\psi =K\psi ,\;\psi \in K$$

with the time-independent Floquet Hamiltonian (or quasienergy operator)

(A4) $$K=-i\hbar \omega \frac{\partial }{\partial \theta }+H\left(\theta \right),$$

defined in an enlarged Hilbert space

(A5) K:=H⊗L,

where $L:=L_{2}\left(S^{1},d\theta /2\pi \right)$ denotes the space of square integrable functions on the circle $S^{1}$ of length 2π, with the scalar product

(A6) $${\langle \xi_{1}|\xi_{2}\rangle }_{L}:=\int_{S^{1}}\frac{d\theta }{2\pi }\;\xi_{1}^{*}\left(\theta \right)\xi_{2}\left(\theta \right).$$

The solution φ(t) is related to the time independent problem as

(A7) $$\varphi \left(x;t;\theta \right)=\psi (x,\theta +\omega \left(t-t_{i}\right);t)$$

with the initial condition in K constructed as the initial condition $\varphi (t_{i})$ of the original problem lifted in the enlarged space as:

(A8) $$\psi \left(x,\theta ;t_{i}\right)=\varphi \left(x;t_{i};\theta \right)⊗{1\;l}_{L}$$

with ${1\;l}_{L}\equiv e^{i(k=0)\theta }$. In the expression φ(x;t;θ), on the left-hand side of (A7), θ is a parameter corresponding to the initial phase of the Hamiltonian, while, in $\psi (x,\theta ;t_{i})$, on the right-hand side, θ is a dynamical variable on the same footing as *x*. The procedure dictated by Equation (A7) to obtain the dynamics in the original space H is as follows:

(i) first, by the use of Equation (A8), lift the initial condition in the enlarged space K keeping θ undetermined;

(ii) next determine ψ(x,θ;t) in K solution of Equation (A3);

(iii) finally replace the angle θ by $\theta +\omega (t-t_{i})$ in ψ and fix the angle θ to the initial phase of the Hamiltonian.

Since *K* is time-independent, the convenient way to compute the solution is to use an eigenfunction expansion

(A9) $$\varphi \left(x;t;\theta \right)=\sum_{\nu }c_{\nu }e^{-i\lambda_{\nu }\left(t-t_{i}\right)/\hbar }\psi_{\nu }\left(x,\theta +\omega \left(t-t_{i}\right)\right)$$

in terms of the eigenelements of *K* (assuming a discrete spectrum)

(A10) $$K\psi_{\nu }\left(x,\theta \right)=\lambda_{\nu }\psi_{\nu }\left(x,\theta \right),$$

with the eigenvalues named quasienergies, with

(A11) $$c_{\nu }={\langle \psi_{\nu }|\varphi \left(t_{i}\right)⊗1\rangle }_{K}={\langle \tilde{\psi_{\nu }}|\varphi \left(t_{i}\right)⊗1\rangle }_{H},$$

where $\tilde{\psi_{\nu }}\left(x\right):=\int_{S^{1}}d\theta /2\pi \;\psi_{\nu }\left(x,\theta \right)$ is the average of $\psi_{\nu }\left(x,\theta \right)$ over the phase, or equivalently, its constant Fourier component.

The Floquet spectrum contains an infinite number of elements, but its eigenelements feature a periodic structure with the index ν being associated to two indices, ν≡n,k:

(A12) $$\psi_{n,k}\left(x,\theta \right)=\psi_{n,0}\left(x,\theta \right)e^{ik\theta },\;\lambda_{n,k}=\lambda_{n,0}+k\hbar \omega ,$$

where the index *n* refers to the molecule’s Hilbert space H (i.e., n=1,⋯,N if $H=C^{N}$), and *k* are all positive or negative integers. This allows one to classify the Floquet eigenstates in families labeled by *n*. The individual members within one family are distinguished by the index *k*. The eigenfunction expansion can be simplified using only one representative of each family (e.g., the one with k=0):

(A13) $$\varphi \left(x;t;\theta \right)=\sum_{n}{\tilde{c}}_{n}\left(\theta \right)e^{-i\lambda_{n,0}\left(t-t_{i}\right)/\hbar }\psi_{n,0}\left(x,\theta +\omega \left(t-t_{i}\right)\right)$$

with

(A14) $${\tilde{c}}_{n}\left(\theta \right):={\langle \psi_{n,0}\left(\theta \right)|\varphi \left(t_{i}\right)\rangle }_{H}.$$

The interpretation of this expansion is as follows: the initial condition is expanded into the set of representatives of each family of eigenvectors $\psi_{n,0}\left(x,\theta \right)$ through the coefficient ${\tilde{c}}_{n}\left(\theta \right)$ (which depends parametrically on θ); during the evolution, the expansion acquires dynamical phases through the eigenvalues $\lambda_{n,0}$ and features a dynamical evolution of θ in the eigenvectors.

### Appendix A.2. Adiabatic Floquet Theory

The adiabatic Floquet theory extends the preceding formulation when the Hamiltonian features an additional non-periodic timescale, which is typically slow with respect to the periodic one. These parameters are gathered under the function r(t) (for instance the time-dependent amplitude of the coupling for the case of a pulse-shaped field and its time-dependent polarization if an interacting polarized laser field is considered), while the non-periodic time-dependence of the phase itself (referred to as chirped pulse) is considered specifically:

(A15) $$H^{r(t)}\left(\theta +\phi (t)+2\pi \right)=H^{r(t)}\left(\theta +\phi (t)\right).$$

The 2π-periodic phase θ+ϕ(t) features an instantaneous (or effective) frequency $\omega \left(t\right)=\dot{\phi }\left(t\right)$, such that $\phi \left(t\right)=\int_{t_{i}}^{t}\omega \left(s\right)ds$, allows one to recover the phase dependence of the previous periodic system when the time of the frequency is frozen.

The state φ(x;t;θ) solution of the Schrödinger equation

(A16) $$i\hbar \frac{\partial }{\partial t}\varphi =H^{r(t)}\left(\theta +\phi (t)\right)\varphi$$

is connected to ψ(x,θ;t) the solution of

(A17) $$i\hbar \frac{\partial }{\partial t}\psi =K^{r(t),\omega (t)}\psi ,$$

with

(A18) $$\omega \left(t\right)=\dot{\phi }\left(t\right),\;\phi \left(t\right)=\int_{t_{i}}^{t}\omega \left(s\right)ds$$

and

(A19) $$K^{r(t),\omega (t)}=H^{r(t)}\left(\theta \right)-i\hbar \omega \left(t\right)\frac{\partial }{\partial \theta }$$

through

(A20) φ(x;t;θ)=ψ(x,θ+ϕ(t);t),

with the initial condition

(A21) $$\psi \left(x,\theta ;t_{i}\right)=\varphi \left(x;t_{i};\theta \right)⊗{1\;l}_{L}.$$

We remark that, at this stage, this correspondence between the original and Floquet dynamics with additional non-periodic time dependent parameters is exact. It does not require a specific number of oscillations of the field in the pulse, nor specific (slow) variation of these parameters.

The physical interpretation of the operator $-i\frac{\partial }{\partial \theta }$ is the relative photon number operator in the limit of large number of photons present in the interacting field. One can thus assign two labels to the eigenstates $|\psi_{n,k}\rangle$, with the label *n* coming from the matter and the label *k* interpreted as the relative photon number which *dresses* the state. A transfer from the initial eigenstate $|\psi_{n,k}^{0}\rangle \equiv |n\rangle ⊗|k\rangle$ with |n〉 a bare state of the quantum system and $|k\rangle \equiv e^{ik\theta }$ a (relative) state of *k* photons *before the field is on*, i.e., before interaction between the matter and the field, to the eigenstate $|\psi_{n^{\prime },k^{\prime }}^{0}\rangle \equiv |n^{\prime }\rangle ⊗|k^{\prime }\rangle$*after the field is off*, i.e., after the interaction, corresponds to the transfer from |n〉 to $|n^{\prime }\rangle$ with the absorption (emission) of $k^{\prime }-k$ photons for negative (positive) $k^{\prime }-k$. When the field is on, the Floquet eigenstates cannot be simply decomposed as a tensor product of a bare matter state and a field state, but are rather an entangled state, as a superposition of such tensored states.

In the limit of slow variations, t≡ϵs with ϵ≪1, one can apply the above formulation with the use the eigenelements of *K*, which depends now on the parameters:

(A22) $$K^{r(t),\omega (t)}\psi_{\nu }^{r(t),\omega (t)}\left(x,\theta \right)=\lambda_{\nu }^{r(t),\omega (t)}\psi_{\nu }^{r(t),\omega (t)}\left(x,\theta \right),$$

on which we derive an adiabatic approximation

(A23) $$\varphi \left(x;t;\theta \right)\approx \sum_{n}{\tilde{c}}_{n}\left(\theta \right)e^{-i\int_{t_{i}}^{t}ds\lambda_{n,k(n)}^{r(s),\omega (s)}/\hbar }\psi_{n,k(n)}^{r(t),\omega (t)}\left(x,\theta +\phi \left(t\right)\right)$$

with

(A24) $${\tilde{c}}_{n}\left(\theta \right):={\langle \psi_{n,k(n)}^{r\left(t_{i}\right),\omega \left(t_{i}\right)}\left(\theta \right)|\varphi \left(t_{i}\right)\rangle }_{H}.$$

Similarly as before, this expansion means that the initial condition is expanded into the set composed of one representative of all the families of eigenvectors $\psi_{n,k(n)}^{r\left(t_{i}\right),\omega \left(t_{i}\right)}\left(x,\theta \right)$ at the initial values of the parameters $r\left(t_{i}\right),\omega \left(t_{i}\right)$ through the coefficient ${\tilde{c}}_{n}\left(\theta \right)$ (which depends parametrically on θ); during the evolution, the expansion acquires dynamical phases through the integration of the eigenvalues $\lambda_{n,0}^{r(t),\omega (t)}$ via the time-dependence of the parameters r(t),ω(t) and features a dynamical evolution of θ and of the parameters in the eigenvectors. The choice of the representatives labelled by k(n) is such that their corresponding eigenvalues are localized in a band of energy of width ℏω defining a *Floquet zone*; the coefficients k(n) depends thus in general on *n*.

A remarkable property is when a single eigenstate is followed by the dynamics, since, then, the resulting single dynamical phase is an irrelevant global phase. Moreover, the control of the dynamics reduces to an analysis of the trajectories of the eigenvalues and, more precisely, to the connection of a trajectory between an initial and a final state [17]. This means that if a state is targeted at a specific time, for instance at the end of the interaction, one just has to drive the dynamics along an adiabatic trajectory, which connects the initial and the desired targeted states; the exact form of the trajectory being unimportant. The dynamics can be thus selective and robust with respect to the precise instantaneous values of the parameters.

The (local) correction of the eigenfunction expansion (A23) is in general O(ϵ). However it can be made in principle exponentially small, $O(e^{-C/\epsilon })$ with *C* a positive constant, with the use of smooth controls and a global analysis of the dynamics [38]. Obstruction of adiabatic passage occurs when the eigenvalues get closer, which can induce non-adiabatic transitions between the eigenstates, as typically given by Landau-Zener avoided crossing. Adiabatic passage can be made more efficient when the eigenvalues are parallel to each other during the dynamics [39,40,41].

## Appendix B. Algorithms

Our numerical simulations were implemented under *Matlab* using the built-in function *fmincon*. This command is dedicated to finding the minimum of a constrained non-linear multivariable function.

Our script is based on three loops as shown in the workflow in Figure A1. The first loop is dedicated to the initialization of the Fourier coefficients of Ω(t) under the constraints given by the RWA π-pulse conditions. The second loop explores a larger space of solutions by removing the RWA π-pulse constraint. Finally the last loop realizes the optimization by adding the detuning.

Note that the condition (5) is imposed in the first two loops by choosing $\Omega_{N}=0$ in the Fourier expansion of Ω(t). In the third loop, this condition (5) is satisfied by the specific form of the phase (36).

## Appendix C. Perturbation Theory Formulated with KAM Techniques

We decompose the Hamiltonian with an unperturbed Hamiltonian $H_{0}$ and a perturbation $\varepsilon V_{1}$:

(A25) $$H=H_{0}+\varepsilon V_{1}.$$

We construct a unitary transformation (so-called KAM or contact transformation) $T_{1}=e^{\varepsilon W_{1}}$, with $W_{1}^{†}=-W_{1}$ such that

(A26) $$e^{-\varepsilon W_{1}}He^{\varepsilon W_{1}}=H_{0}+\varepsilon D_{1}+\varepsilon^{2}V_{2},$$

where $D_{1}$ is a diagonal operator, i.e., satisfying $[H_{0},D_{1}]=0$. Thus the perturbation is reduced from order ε to order $\varepsilon^{2}$.

In order to determine $D_{1}$ and $W_{1}$, one expands Equation (A26) in powers of ε and require that the terms of order ε cancel out:

$$\begin{array}{cc} e^{-\varepsilon W_{1}}He^{\varepsilon W_{1}} & =\left(1\;l-\varepsilon W_{1}+\frac{1}{2}\varepsilon^{2}W_{1}^{2}+\cdots \right)\left(H_{0}+\varepsilon V_{1}\right)\left(1\;l+\varepsilon W_{1}+\frac{1}{2}\varepsilon^{2}W_{1}^{2}+\cdots \right)\\ \; & =H_{0}+\varepsilon \left(\left[H_{0},W_{1}\right]+V_{1}\right)+\frac{1}{2}\varepsilon^{2}\left[\left[H_{0},W_{1}\right],W_{1}\right]+\varepsilon^{2}\left[V_{1},W_{1}\right]+\cdots \end{array}$$

(A27) $$\;=H_{0}+\varepsilon D_{1}+\frac{1}{2}\varepsilon^{2}\left[V_{1},W_{1}\right]+\frac{1}{2}\varepsilon^{2}\left[D_{1},W_{1}\right]+\cdots$$

This leads to the two equations

(A28a) $$\begin{array}{ccc} \left[H_{0},W_{1}\right]+V_{1}-D_{1} & = & 0, \end{array}$$

(A28b) $$\begin{array}{ccc} {}[H_{0},D_{1}] & = & 0, \end{array}$$

and the first terms of the resulting second order perturbation read:

(A29) $$\begin{array}{c} \varepsilon^{2}V_{2}=\frac{\varepsilon^{2}}{2}\left[V_{1},W_{1}\right]+\frac{\varepsilon^{2}}{2!}\left[D_{1},W_{1}\right]+\ldots \end{array}$$

The solution of Equation (A28) can be given using the eigenvalues and eigenvectors of $H_{0}$ which we will denote by $\lambda_{\nu }^{0}$ and |ν,j〉 (we use an index ν that labels the different eigenvalues, and *j* distinguishes different basis vectors corresponding to a degenerate eigenvalue). We define a projection operator $\Pi_{H_{0}}$ that extracts from the perturbation $V_{1}$ the diagonal component with respect to the eigenbasis of $H_{0}$:

(A30) $$\Pi_{H_{0}}V_{1}\equiv {\bar{V}}_{1}=\sum_{\nu ,j,j^{\prime }}\left|\nu ,j\rangle \langle \nu ,j\right|V_{1}|\nu ,j^{\prime }\rangle \langle \nu ,j^{\prime }|.$$

With this notation a solution of (A28) can be written as

(A31a) $$\begin{array}{ccc} D_{1} & = & {\bar{V}}_{1}=\mathrm{diagonal}\;\mathrm{part}\;\mathrm{of}\;V_{1}, \end{array}$$

(A31b) $$\begin{array}{ccc} W_{1} & = & -\sum_{\nu ,j,j^{\prime },\nu^{\prime }\neq \nu }\frac{\left|\nu ,j\rangle \langle \nu ,j\right|V_{1}|\nu^{\prime },j^{\prime }\rangle \langle \nu^{\prime },j^{\prime }|}{\lambda_{\nu }^{0}-\lambda_{\nu^{\prime }}^{0}}. \end{array}$$

$D_{1}$ is interpreted as the averaging of $V_{1}$ with respect to $H_{0}$. The perturbation theory does not apply when a resonance appears, which is detected by a degeneracy $\lambda_{\nu }^{0}=\lambda_{\nu^{\prime }}^{0}$, leading to a zero denominator of $W_{1}$.

The KAM transformations can be iterated. One usually applies another KAM transformation consisting in extracting the averaging of $V_{2}$, and the effective Hamiltonian of second order reads

(A32) $$\begin{array}{c} H_{\mathrm{eff}}=H_{0}+\varepsilon {\bar{V}}_{1}+\varepsilon^{2}{\bar{V}}_{2}. \end{array}$$

## Appendix D. High-Frequency Perturbation Theory

High frequency perturbation theory applies to situations in which the frequency ω of the perturbation is high with respect to the internal frequencies of the considered system. We formulate with a Floquet Hamiltonian of the form

(A33) $$K=-i\hbar \omega \frac{\partial }{\partial \theta }+H_{0}+V_{1}\left(\theta \right).$$

Since we are interested in the limit ℏω→∞, we define a small parameter ϵ:=1/(ℏω) and we rewrite

(A34) $$K=\hbar \omega \hat{K}$$

with

(A35) $$\hat{K}=-i\frac{\partial }{\partial \theta }+\epsilon \left(H_{0}+V_{1}\right).$$

The difference with the preceding section is that here $H_{0}$ and *V* are both of order ϵ. Thus we take as the unperturbed Floquet Hamiltonian just

(A36) $${\hat{K}}_{0}:=-i\frac{\partial }{\partial \theta }.$$

If the frequency is large compared with the frequencies of the system, there will not be any resonances. We can thus proceed with the perturbative KAM transformation: $e^{\epsilon W_{1}\left(\theta \right)}$, with $W_{1}^{†}=-W_{1}$ such that

(A37) $$e^{-\epsilon W_{1}}\hat{K}e^{\epsilon W_{1}}={\hat{K}}_{0}+\epsilon D_{1}+\epsilon^{2}V_{2},$$

where $D_{1}$ is a θ-independent operator such that $[{\hat{K}}_{0},D_{1}]=0$. The generator $W_{1}$ of the contact transformation is determined by the equations

(A38a) $$\begin{array}{ccc} \left[K_{0},W_{1}\right]+H_{0}+V_{1}-D_{1} & = & 0, \end{array}$$

(A38b) $$\begin{array}{ccc} \left[K_{0},D_{1}\right] & = & 0. \end{array}$$

Equation (A38a) can be written as

(A39) $$-i\frac{\partial W_{1}}{\partial \theta }+H_{0}+V_{1}-D_{1}=0,$$

whose general solution is given by

(A40) $$W_{1}=-i\int^{\theta }d\theta \;\left(H_{0}+V_{1}-D_{1}\right),$$

where we have removed an arbitrary constant. Since $W_{1}\left(\theta \right)$ is a multiplication operator acting on functions of the angle θ, it must be necessarily 2π-periodic. This condition determines $D_{1}$ uniquely in terms of averaging

(A41) $$\bar{V_{1}}:=\int_{0}^{2\pi }\frac{d\theta }{2\pi }V_{1}\left(x,\theta \right)$$

as

(A42) $$D_{1}=H_{0}+\bar{V_{1}}.$$

Thus we obtain

(A43) $$W_{1}\left(\theta \right)=-i\int^{\theta }d\theta \;\left(V_{1}\left(\theta \right)-\bar{V_{1}}\right).$$

The remaining perturbation of order $\epsilon^{2}$ can be written as

(A44) $$\epsilon^{2}V_{2}=\frac{\epsilon^{2}}{2!}\left[V_{1}+\bar{V_{1}}+2H_{0},W_{1}\right]+\epsilon^{3}\ldots ,$$

We can apply a second contact transformation $e^{\epsilon^{2}W_{2}}$ (with respect to ${\hat{K}}_{0}$), $W_{2}=-i\int^{\theta }d\theta \;\left(V_{2}-\bar{V_{2}}\right)$, which averages this rest (A44) with respect to θ and leads to correction of order $\epsilon^{3}$. We thus obtain the effective high frequency Hamiltonian $H^{\mathrm{HF}}$ (independent of θ) of second order

(A45) $$H^{\mathrm{HF}}=H_{0}+\bar{V_{1}}+\epsilon \bar{V_{2}}.$$

As an example, one considers an Hamiltonian of the form:

(A46) $$\begin{array}{c} H=\frac{1}{2}\Omega_{0}\left[\begin{array}{cc} 0 & 1\\ 1 & 0 \end{array}\right]+\frac{1}{2}\Omega_{-2}\left[\begin{array}{cc} 0 & e^{-2i\omega t}\\ e^{2i\omega t} & 0 \end{array}\right]+\frac{1}{2}\Omega_{2}\left[\begin{array}{cc} 0 & e^{2i\omega t}\\ e^{-2i\omega t} & 0 \end{array}\right], \end{array}$$

i.e.,

(A47) $$\begin{array}{c} \hat{K}=\frac{K}{\omega }=-i\frac{\partial }{\partial \theta }+\frac{1}{2}\frac{\Omega_{0}}{\omega }\left[\begin{array}{cc} 0 & 1\\ 1 & 0 \end{array}\right]+\frac{1}{2}\frac{\Omega_{-2}}{\omega }\left[\begin{array}{cc} 0 & e^{-2i\theta }\\ e^{2i\theta } & 0 \end{array}\right]+\frac{1}{2}\frac{\Omega_{2}}{\omega }\left[\begin{array}{cc} 0 & e^{2i\theta }\\ e^{-2i\theta } & 0 \end{array}\right], \end{array}$$

where

(A48) $$\begin{array}{c} \epsilon H_{0}\equiv \frac{1}{2}\frac{\Omega_{0}}{\omega }\left[\begin{array}{cc} 0 & 1\\ 1 & 0 \end{array}\right],\;\epsilon V_{1}\equiv \frac{1}{2}\frac{\Omega_{-2}}{\omega }\left[\begin{array}{cc} 0 & e^{-2i\theta }\\ e^{2i\theta } & 0 \end{array}\right]+\frac{1}{2}\frac{\Omega_{2}}{\omega }\left[\begin{array}{cc} 0 & e^{2i\theta }\\ e^{-2i\theta } & 0 \end{array}\right], \end{array}$$

in the high frequency limit, rewritten with the dimensionless coefficients $\Omega_{-2}/2\omega \to 0$, $\Omega_{2}/2\omega \to 0$, $\Omega_{0}/2\omega \to 0$. We obtain ${\bar{V}}_{1}=0$,

(A49) $$W_{1}\left(\theta \right)=\frac{1}{4}\Omega_{-2}\left[\begin{array}{cc} 0 & e^{-2i\theta }\\ -e^{2i\theta } & 0 \end{array}\right]+\frac{1}{4}\Omega_{2}\left[\begin{array}{cc} 0 & -e^{2i\theta }\\ e^{-2i\theta } & 0 \end{array}\right],$$

and ${\bar{V}}_{2}=\frac{1}{2}\bar{\left[V_{1},W_{1}\right]}+\bar{\left[H_{0},W_{1}\right]}+\ldots ,$ where

(A50) $$\bar{\left[H_{0},W_{1}\right]}=0,\;\bar{\left[V_{1},W_{1}\right]}=\frac{1}{4}\left(\Omega_{-2}^{2}-\Omega_{2}^{2}\right)\left[\begin{array}{cc} -1 & 0\\ 0 & 1 \end{array}\right],$$

i.e., at the second order

(A51) $${\bar{V}}_{2}=\frac{1}{8}\left(\Omega_{-2}^{2}-\Omega_{2}^{2}\right)\left[\begin{array}{cc} -1 & 0\\ 0 & 1 \end{array}\right].$$

The high-frequency effective Hamiltonian reads then

(A52) $$H^{\mathrm{HF}}=\frac{1}{2}\left[\begin{array}{cc} -\frac{\Omega_{-2}^{2}-\Omega_{2}^{2}}{4\omega } & \Omega_{0}\\ \Omega_{0} & \frac{\Omega_{-2}^{2}-\Omega_{2}^{2}}{4\omega } \end{array}\right].$$

The diagonal term can be neglected when

(A53) $$\Omega_{0}\gg \frac{1}{2}\frac{|\Omega_{-2}^{2}-\Omega_{2}^{2}|}{\omega }.$$

If we consider a time-dependent Ω(t), the transformation $T_{1}=e^{\epsilon W_{1}}$ introduces in the Schrödinger equation an additional term of the form:

(A54) $$-i\epsilon T^{†}\dot{T}=-i\epsilon^{2}{\dot{W}}_{1}=-i\frac{\dot{\Omega }}{4\omega^{2}}\left[\begin{array}{cc} 0 & e^{-2i\theta }\\ -e^{2i\theta } & 0 \end{array}\right],$$

which should be treated perturbatively like $\epsilon V_{1}$. It introduces an additional term in the diagonal of (A52):

(A55) $$H^{\mathrm{HF}}=\frac{1}{2}\left[\begin{array}{cc} -\frac{\Omega_{-2}^{2}-\Omega_{2}^{2}}{4\omega }+\frac{{\dot{\Omega }}_{-2}^{2}-{\dot{\Omega }}_{2}^{2}}{16\omega^{3}} & \Omega_{0}\\ \Omega_{0} & \frac{\Omega_{-2}^{2}-\Omega_{2}^{2}}{4\omega }-\frac{{\dot{\Omega }}_{-2}^{2}-{\dot{\Omega }}_{2}^{2}}{16\omega^{3}} \end{array}\right].$$
